# Loss of Ifnar1 in Pancreatic Acinar Cells Ameliorates the Disease Course of Acute Pancreatitis

**DOI:** 10.1371/journal.pone.0143735

**Published:** 2015-11-30

**Authors:** Katharina J. Miller, Susanne Raulefs, Bo Kong, Katja Steiger, Ivonne Regel, Andreas Gewies, Jörg Kleeff, Christoph W. Michalski

**Affiliations:** 1 Department of Surgery, Technische Universität München, Munich, Germany; 2 Institute of Pathology, Technische Universität München, Munich, Germany; 3 Institute für Klinische Chemie und Pathobiochemie, Technische Universität München, Munich, Germany; 4 Department of Surgery, University Hospital Heidelberg, Heidelberg, Germany; Centro Nacional de Investigaciones Oncológicas (CNIO), SPAIN

## Abstract

Type I interferon constitutes an essential component of the combinational therapy against viral disease. Acute pancreatitis is one side effect of type I interferon-based therapy, implying that activation of type I interferon signaling affects the homeostasis and integrity of pancreatic acinar cells. Here, we investigated the role of type I interferon signaling in pancreatic acinar cells using a caerulein-induced murine model of acute pancreatitis. Pancreas-specific ablation of interferon (alpha and beta) receptor 1 (Ifnar1) partially protected animals from caerulein-induced pancreatitis, as demonstrated by reduced tissue damage. Profiling of infiltrating immune cells revealed that this dampened tissue damage response correlated with the number of macrophages in the pancreas. Pharmacologic depletion of macrophages reversed the protective effect of Ifnar1 deficiency. Furthermore, expression of chemokine (C-C motif) ligand 2 (Ccl2), a potent factor for macrophage recruitment, was significantly increased in the Ifnar1-deficient pancreas. Thus, type I interferon signaling in pancreatic acinar cells controls pancreatic homeostasis by affecting the macrophage-mediated inflammatory response in the pancreas.

## Introduction

Type I interferons belong to the cytokine signaling molecules and protect the host by exhibiting an anti-viral and anti-tumor function [1–3]. Type I interferon constitutes an essential component of the combinational therapy against viral disease and cancer [4, 5]. Notably, acute pancreatitis is reported to be a relevant side effect of such type I interferon-based therapies [6–9] implying that activation of type I interferon signaling affects the physiological homeostasis of the pancreas.

In the disease course of acute pancreatitis, pancreatic acinar cells have been shown to release chemokines [10, 11] and cytokines such as tumor necrosis factor, interleukins and interferons [12–14], which are key drivers of the inflammation [15]. Cytokines are known to activate monocytes and lead to a polarization of these cells into macrophages [16–18], whereas chemokines act as chemotactic guides to recruit macrophages to the inflamed pancreas [19]. In this regard, chemokine (C-C motif) ligand 2 (Ccl2) is one of the most important factors for the recruitment of macrophages [20]. During inflammation, macrophages support the clearance of the destroyed tissue through phagocytosis of damaged and dead cells [21] and promote regeneration by supporting the resolution of inflammation, tissue renewal and angiogenesis [22, 23].

As the type I interferon-driven Ccl2 production is responsible for the recruitment of macrophages in other inflammatory contexts [24, 25], we hypothesized that type I interferon signaling affects the disease course of acute pancreatitis by interfering with the macrophage-mediated inflammatory response by creating a macrophage-priming environment. To assess this, we used a caerulein-induced pancreatitis model and investigated the influence of restriction of type I interferon signaling in pancreatic acinar cells on the development of acute pancreatitis.

## Materials and Methods

### Experimental animals

C57-Bl6/J mice (WT mice) were purchased from Janvier, France. Ifnar^fl/fl^ mice were a kind gift of Prof. Dr. U. Kalinke from the Paul-Ehrlich-Institute (Langen, Germany). Ifnar^fl/fl^ mice were crossed with Ptf1a^Cre/+^ mice to generate Ptf1a^Cre/+^; Ifnar^fl/fl^ (Ifnar^del^) mice. Mice were bred at the specific pathogen-free mouse facility at the Technical University of Munich and fed a normal chow diet (Altromin, number: 1324) and water *ad libitum*. Environmental conditions were a room temperature of 20–22°C, a 12:12 light dark cycle with lights on at 7 am and off at 7 pm, an air humidity of 50% and an air pressure of 50 Pa. The animals housed in groups of 5 animals (>30g/mouse) per cage from the type II IVC blueline long from Tecniplast with dimensions of 35 cm l x14 cm w x13 cm h. Every cage was equipped with bedding (Lignocel select fine), a red hut (BIOSCAPE EBECO, Castrop-Rauxel, Germany) and nesting material. Based on daily monitoring and hygienic conditions bedding was changed once per week. During housing, animals were monitored daily for health status. Before the experiments the animals were weighed mean ± SD 21,8 ± 2,9 grams. Experimental mice were age-matching and used at 8–10 weeks of age (n = 3–9). Euthanasia was performed under Isofluran (CP Pharma, Burgdorf, Germany) anesthesia through cervical dislocation. No adverse events were observed during housing or experiments. All mouse strains were on C57-Bl6/J background. All mouse experiments and procedures were approved by the Institutional Animal Care and Use Committees of the Technical University of Munich (reference number: 55.2-1-54-2532-116-2013). All procedures were in accordance with the Office of Laboratory Animal Welfare and the German Federal Animal Protection Laws.

All sections of this report adhere to the ARRIVE Guidelines for reporting animal research.

### Caerulein-induced acute pancreatitis

Acute pancreatitis was induced in at least three to nine 8–10 weeks old male and female WT and Ifnar^del^ mice. The mice received intraperitoneal injections of caerulein (Sigma-Aldrich, Steinheim, Germany) eight times a day hourly for two consecutive days with a total of 100 μg/kg body weight per mouse. The final day of caerulein injection was considered as day 0. For alleviation of pain, caerulein treated mice received subcutaneously Buprenodale (Dechra, Albrecht GmbH, Aulendorf, Germany) in a concentration of 0,1 mg/kg body weight per mouse simultaneously with the first caerulein injection, respectively.

### In vivo depletion of macrophages

For the specific depletion of macrophages, at least three to five 8–10 week old male and female WT and Ifnar^del^ mice were treated with 5 mg/kg bodyweight of either Clodronate or PBS filled liposomes (ClodronateLiposomes.org, Amsterdam, The Netherlands,) per mouse [26]. Liposomes were administrated once a day every second day by intraperitoneal injection. The liposome treatment started four days before the first caerulein injection and was continued till the end of the individual experiments. The depletion efficiency was confirmed by immunohistochemical staining of macrophages in the pancreata of treated mice.

### Histological analysis

Pancreatic damage and regeneration was quantified by scoring one slide per mouse with a whole tissue section for at least three to nine mice per group. Proportion of normal pancreas and acinar-to-ductal-metaplasia were specified in percent [%] of the whole pancreatic tissue. Edema, necrosis, grade and distribution of pancreatitis were scored (0–3). Details of the scoring system were listed in S1 Table. The evaluation of the inflammation grade was geared to the inflammation grading score of Schmidt el al. [27]. Total pancreatitis was calculated (grade of inflammation x distribution of inflammation). The scoring was designed and performed in a blinded manner by an experienced pathologist.

### Immunohistochemistry

Pancreata of at least three to nine mice per group were fixed in 4% PFA/PBS for 12–16 hours, dehydrated, paraffin embedded, cut into 2.5–3 μm thick sections, placed on slides and dried at 37°C overnight. Antigen retrieval was performed by pretreatment of the slides in citrate buffer (pH 6.0; 10 mM Citric Acid, 0.05% Tween 20) in a microwave oven for 10–30 min. Endogenous peroxidase activity was quenched by incubation in methanol containing 3% hydrogen peroxide at room temperature for 3–5 min. After blocking of nonspecific reactivity with TBS (pH 7.4; 0.1 M Tris Base, 1.4 M NaCl) containing 3% BSA or 3% BSA with Avidin, sections were incubated with the respective antibody at 4°C overnight followed by incubation with horseradish peroxidase-linked antibodies for 1 h at room temperature. Subsequent color reaction was performed with diaminobenzidine (Dako, Hamburg, Germany) and counterstaining with Mayer’s hematoxylin (Merck-Millipore, Darmstadt, Germany). The following primary antibodies were used: rat α-CD45 (1:10; BD, Heidelberg, Germany), rat α-F4/80 (BM8) (1:160; abcam, Cambridge, UK), rabbit α-myeloperoxidase (ready-to-use; CellMarque, California, USA), rabbit α-CD3 (1:100; abcam, Cambridge, UK), and rat α-B220 (1:500; R&D Systems). The following secondary antibodies were used: rabbit HRP (horseradish peroxidase)- labelled α-rat IgG (1:50; Dako, Hamburg, Germany), goat HRP-labelled polymer α-mouse (ready-to-use; Dako, Hamburg, Germany) and goat HRP-labelled polymer α-rabbit (ready-to-use; Dako, Hamburg, Germany).

### RNA isolation

For the analysis of expression levels of different genes, total mRNA was isolated from whole pancreatic tissues of three to seven caerulein treated and untreated WT and Ifnar^del^ mice, respectively, with the RNeasy Mini Kit (Qiagen). Tissue was harvested 4 h after the last injection. Reverse transcription was performed with the RevertAid Reverse Transcriptase kit (Thermo Scientific) according to manufacturer's instructions.

### Quantitative Real-Time Polymerase Chain Reaction

Quantitative real time PCR (qRT PCR) was carried out using the LightCycler^TM^480 system with the SYBR Green 1 master kit (Roche diagnostics). Expression of the target gene was normalized to the mouse housekeeping gene *Ppib* (Peptidyl-prolyl cis-trans isomerase B). Primer sequences will be provided upon request.

### Serum Analysis

For serum analyses blood from three to seven mice per group was incubated at room temperature for 30 min and then centrifuged (5000 g, 20 min, 20°C) in order to separate blood cells and any other solid material from the liquid. Serum analysis for blood serum levels of amylase, lipase, LDH and calcium were performed by the Department of Clinical Chemistry of the Klinikum rechts der Isar of the Technical University Munich.

### Statistics

All data are expressed as mean ± SD. Statistical calculations were performed using GraphPad Prism 5 (GraphPad Software Inc.). Unpaired Student’s t-test for n>10 or Mann-Whitney test for n<10 were used, as appropriate. *P*-values <0.05 were considered as being statistically significant.

## Results

### No obvious differences between WT and Ifnar^del^ mice

**Fig 1:**
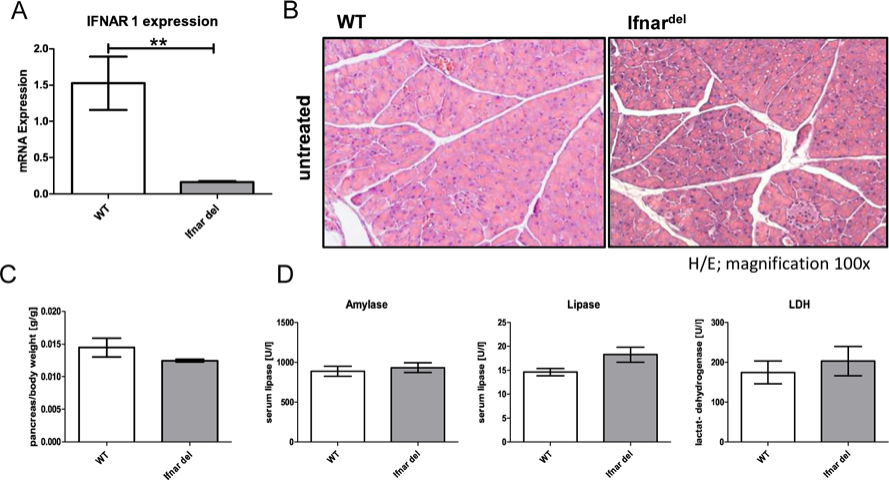
Characterization of untreated Ifnar^del^ mice reveals no differences to wild type mice. (A) qRT PCR analysis of mRNA levels of Ifnar1 from whole pancreatic tissue of untreated WT and Ifnar^del^ mice. (n = 3 per group. Bars indicate mean +/- SD. Normalized on the mRNA of the housekeeping gene *Ppib*. **P<0.01, Mann-Whitney-test). (B) Representative H&E staining of the pancreas from 8 week old WT and Ifnar^del^ mice without treatment (original magnification, 100x). (C) Pancreas weight/body weight ratio of 8 week old WT and Ifnar^del^ mice without treatment (n = 7 per group. Bars indicate mean +/- SD). (D) Amylase, lipase and LDH (lactat-dehydrogenase) levels in the serum from of 8 week old WT and Ifnar^del^ mice without treatment (n = 7 per group. Bars indicate mean +/- SD).

Ifnar^del^ mice have a knockout of the type I interferon receptor subunit 1 restricted to epithelial cells of the pancreas which leads to an interruption of the interferon alpha receptor expression (Fig 1A). The histological comparison of untreated eight weeks old C57-BL6/J (wild type/WT) mice with Ifnar^del^ mice revealed no obvious differences between the two genotypes. H/E staining of WT and Ifnar^del^ mice showed intact acinar lobes without any other histological abnormalities in the structure of the pancreas (Fig 1B). The body weight as well as the pancreas weight of WT mice corresponded to that of Ifnar^del^ mice (S1 Fig). Further, the pancreas/body weight ratio was not significantly different between WT and Ifnar^del^ mice (Fig 1C). Analysis of the blood serum levels of untreated WT and Ifnar^del^ mice indicated comparable levels of amylase, lipase and LDH (Fig 1D).

### Caerulein-induced pancreatitis is focally restricted in Ifnar^del^ mice

**Fig 2:**
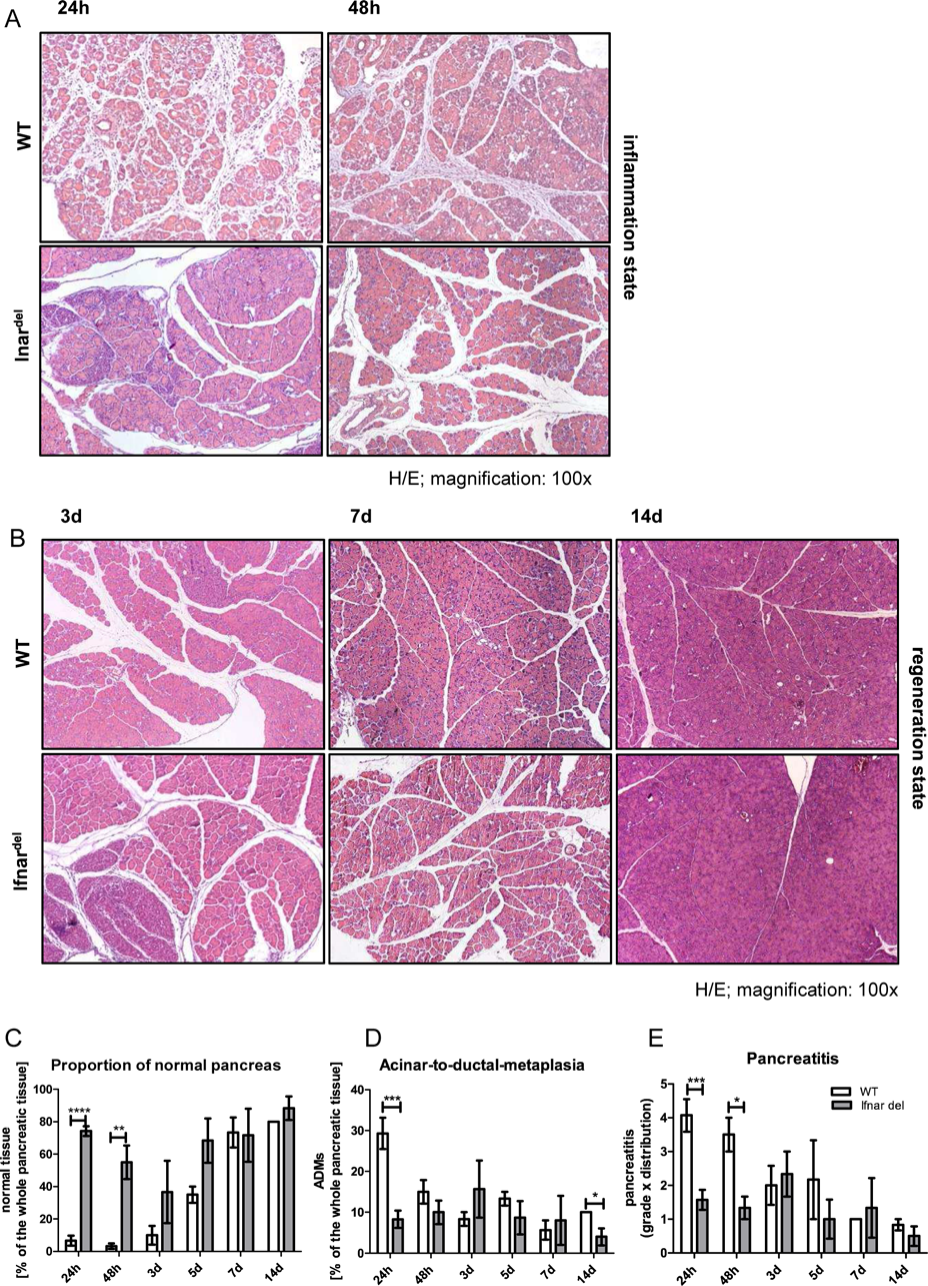
Pancreatic inflammation after caerulein- induced injury is limited in Ifnar^del^ mice. (A) Representative H&E staining of the pancreas from WT and Ifnar^del^ mice, 24 and 48 hours following caerulein-induced injury (original magnification, 100x). (B) Representative H&E staining of the pancreas from WT and Ifnar^del^ mice, 3, 7 and 14 days following caerulein-induced injury (original magnification, 100x). (C-E) Combined and individual scoring of pancreatic histological parameters from WT and Ifnar^del^ mice, 24, 48 hours, 3, 5, 7 and 14 days following caerulein-induced injury (n = 3–9 per group. Bars indicate mean +/- SD. *P<0.05, **P<0.01, ***P<0.001, ****P<0.0001, Mann-Whitney-test).

In WT mice, treatment with the cholecystokinin analog caerulein leads to pancreatitis [28, 29] concomitant with the activation of the type I interferon signaling (S2 Fig) To address the consequences of interferon signaling disruption during the inflammation and regeneration process we treated mice with caerulein to induce a pancreatitis in WT and Ifnar^del^ mice [30]. The natural course of acute pancreatitis and the regenerative process consists of three distinctive and transient phases of inflammation, metaplasia and redifferentiation [31], last two summarized as regeneration. Within the inflammation phase (24–48 h after the last caerulein injection), WT mice underwent a broad tissue damage with a de-granulation of acini with immune cells infiltration. In contrast, Ifnar^del^ mice exhibited less infiltrating immune cells and the pancreas was composed of normal, undamaged tissue and only scattered inflammatory areas were visible (Fig 2A). During the regeneration phase (3–14 d after the last caerulein injection) WT as well as Ifnar^del^ mice completely regenerated. After 14 days, the exocrine compartment of WT and Ifnar^del^ mice revealed a histological structure similar to the untreated counterparts (Fig 1B and Fig 2B). To quantify and evaluate the damage and changes of the pancreatic tissue during inflammation and regeneration, we developed a specific scoring system that characterizes different parameters of pancreatitis and pancreatitis related processes (S1 Table). The proportion of undamaged pancreas in WT mice was low during the inflammation phase and increased through the regeneration whereas in Ifnar^del^ mice the level of intact tissue was consistently high (Fig 2C). Acinar-to-ductal-metaplasia (ADM) in WT mice was prominent directly after the induced injury but transient. Within 7 days, the number of ADMs decreased and the dedifferentiated cells seemed to convert back into acinar cells [32]. In contrast, the dedifferentiated state in Ifnar^del^ mice stayed moderate during the inflammation and regeneration process (Fig 2D). No differences between the two genotypes could be observed in the analysis of edema and necrosis (S3 Fig). The assessment of the pancreatitis was based on the calculation of the grade with the distribution of the inflammation (pancreatitis = grade*distribution of inflammation). WT mice displayed strong pancreatitis which decreased during the regeneration of the organ. In contrast, Ifnar^del^ mice developed a mild pancreatitis but a comparable level of the disease after 3 days of injury (Fig 2E). These data show that Ifnar^del^ mice generated a focally restricted inflammation in regard to the distribution of the inflammation compared to WT mice. Nevertheless, the inflamed areas referred to the grade of inflammation were comparable between WT and Ifnar^del^ mice (S3 Fig).

### Altered immune cell composition with increased macrophage infiltration in Ifnar^del^ mice

**Fig 3:**
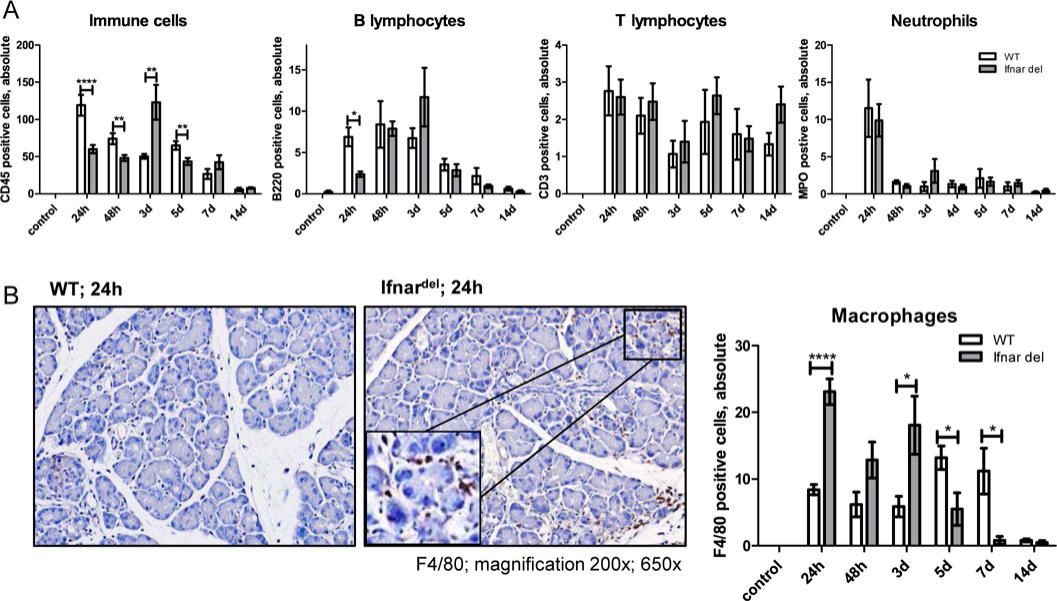
Macrophages are the predominant immune cells infiltrating the pancreas following caerulein-induced injury in Ifnar^del^ mice. (A) The absolute number of CD45-positive immune cells, B220-positive B-lymphocytes, CD3-positive T-lymphocytes and MPO-positive neutrophils was counted in five separate high power fields for each section of untreated WT and Ifnar^del^ mice and 24, 48 hours, 3, 5, 7 and 14 days following caerulein-induced injury (n = 3–9 per group. Bars indicate mean +/- SD. *P<0.05, **P<0.01, ****P<0.0001, Student´s t-test). (B) Immunohistochemical staining for F4/80-positive macrophages in the pancreas from WT and Ifnar^del^ mice 24h following caerulein-induced injury and counting of the absolute number of positive cells in five separate high power fields for each section of untreated WT and Ifnar^del^ mice and 24, 48 hours, 3, 5, 7 and 14 days following caerulein-induced injury (n = 3–9 per group. Bars indicate mean +/- SD. *P<0.05, ****P<0.0001, Student´s t-test. Original magnification, 200x and 650x).

Next, we analyzed the number and composition of infiltrating cells in untreated WT and Ifnar^del^ mice and during the inflammation and regeneration phase. Staining of the common immune cell marker CD45 showed augmented recruitment of immune cells in WT mice in comparison to Ifnar^del^ mice (Fig 3A). Specific staining for B220-positive B-lymphocytes, CD3-positive T-lymphocytes and myeloperoxidase-positive neutrophils revealed no significant differences in the distribution of these immune cells between WT and Ifnar^del^ mice (Fig 3A). Interestingly, there were striking differences in the quantity of F4/80-positive macrophages during the inflammation and regeneration process between WT and Ifnar^del^ mice. WT mice displayed scarce infiltration of macrophages during the inflammation phase (24–48 h) and early regeneration phase (3 d) with an increase in the number of cells in the later regeneration phase (5–7 d) (Fig 3B). In contrast, macrophages were the predominant immune cell type in the pancreas of Ifnar^del^ mice. During the inflammation phase (24–48 h) and the first phase of the regeneration (3 d) there were significantly more F4/80-positive cells in Ifnar^del^ compared to WT mice. During the later regeneration phase, the number of macrophages decreased in Ifnar^del^ mice (Fig 3B). 14 days after tissue injury, there were equally low numbers of macrophages in both genotypes. These data indicate that limitation of type 1 interferon signaling in acinar cells of the pancreas results in aberrant immune cell recruitment after caerulein- induced injury.

### Depletion of macrophages rescues the focally restricted inflammation phenotype

**Fig 4:**
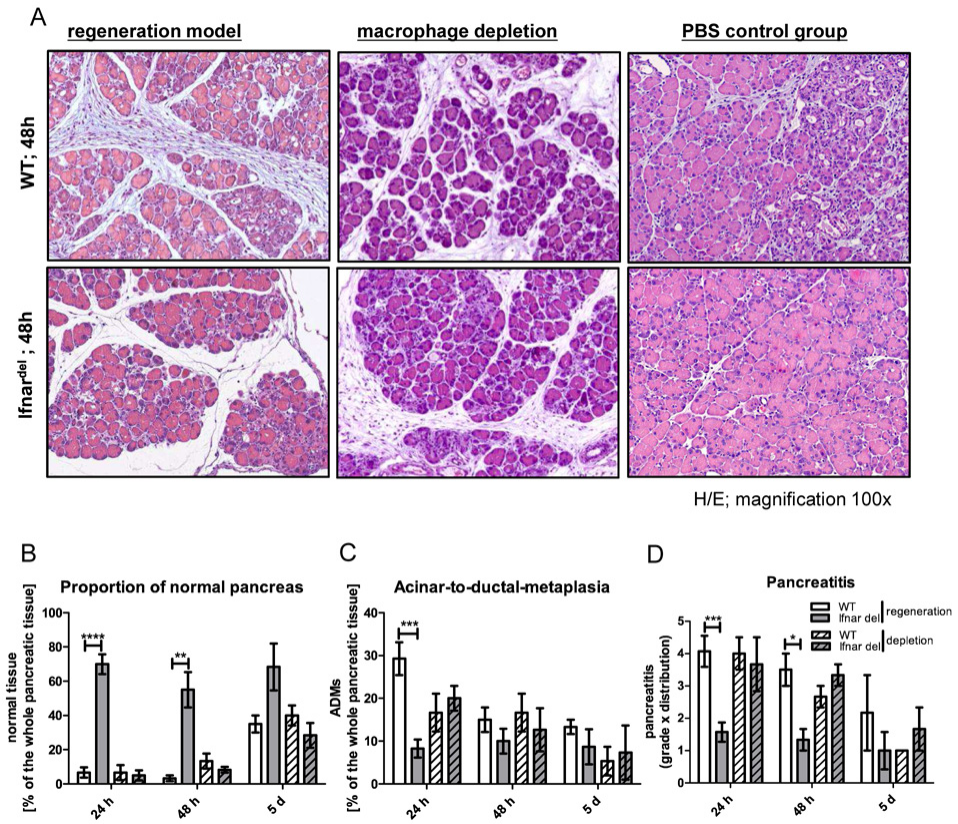
Depletion of macrophages rescues the focally restricted inflammation phenotype in Ifnar^del^ mice. (A) Representative H&E staining of the pancreas from WT and Ifnar^del^ mice 48 hours after treatment. Mice were either treated with caerulein (regeneration model, left), caerulein plus clodronate filled liposomes (macrophage depletion, middle) or caerulein plus PBS filled liposomes (PBS control group, right) as described in the methods section (n = 3; original magnification, 100x). (B-D) Combined and individual scoring of pancreatic histological parameters from WT and Ifnar^del^ mice 24 and 48 hours, and 5 days following caerulein-induced injury (plain bars) or caerulein-induced injury combined with macrophage depletion (striped bars) (n = 3–9 per group. Bars indicate mean +/- SD. *P<0.05, **P<0.01, ***P<0.001, ****P<0.0001, Mann-Whitney-test).

Macrophages are recruited in the late regeneration phase after pancreatic injury (3–5 d) in WT mice (Fig 3B). To analyze the impact of the macrophage infiltration in Ifnar^del^ mice in the early inflammation phase we depleted macrophages using clodronate liposomes [26]. Depletion was confirmed by staining for the macrophage marker F4/80 revealing a complete elimination of macrophages in the pancreas (S4 Fig). Depletion of the macrophages had no effect on the inflammatory response in WT mice (Fig 4). Interestingly, clodronate treated Ifnar^del^ mice exhibited a distinct immune cell infiltration and underwent extensive tissue damage with a de-granulation of acinar cells compared to only caerulein treated Ifnar^del^ mice. (Fig 4A, lower row, 48 h; Fig 4B). The formation of ADMs in Ifnar^del^ mice was comparable to WT mice (Fig 4C). Moreover, in the absence of macrophages, Ifnar^del^ mice revealed comparable pancreatitis as in WT mice (Fig 4D). Furthermore, the amount and the composition of immune cells revealed a similar distribution of CD45-positive immune cells, T- and B- lymphocytes as well as neutrophils (S5 Fig). Treatment with PBS filled liposomes, served as vesicle controls, revealed no differences to the regeneration model analyzing WT as well as Ifnar^del^ mice, respectively (Fig 4A). Thus, infiltrating macrophages might protect the pancreas against the onset of a severe pancreatitis in Ifnar^del^ mice.

### Proinflammatory cytokine expression in WT and Ifnar^del^ mice

**Fig 5:**
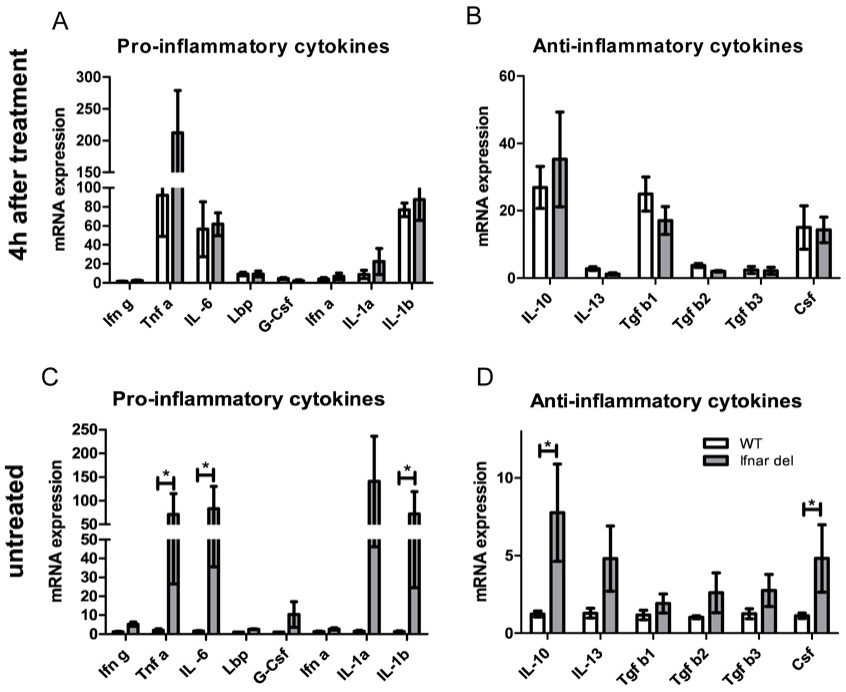
Proinflammatory cytokine expression in WT and Ifnar^del^ mice. (A-B) qRT PCR analysis of mRNA levels of the proinflammatory cytokines *Ifnγ*, *Tnfα*, *IL-6*, *Lbp*, *G-Csf*, *Ifnα*, *IL-1α* and *IL-1β* (A) and the anti-inflammatory *cytokines IL-10*, *IL-13*, *Tgfβ1–3* and *Csf* (B) from whole pancreatic tissue of WT and Ifnar^del^ mice harvested 4 hours following caerulein-induced injury (n = 3 per group. Bars indicate mean +/- SD. Normalized on the mRNA of the housekeeping gene *Ppib*). (C-D) qRT PCR analysis of mRNA levels of the proinflammatory cytokines *Ifnγ*, *Tnfα*, *IL-6*, *Lbp*, *G-Csf*, *Ifnα*, *IL-1α* and *IL-1β* (C) and the anti- inflammatory cytokines *IL-10*, *IL-13*, *Tgfβ1–3* and *Csf* (D) from whole pancreatic tissue of untreated WT and Ifnar^del^ mice (n = 7 per group. Bars indicate mean +/- SD. Normalized on the mRNA of the housekeeping gene *Ppib*. *P<0.05, unpaired Student´s t-test).

To investigate whether the aberrant macrophage infiltration in Ifnar^del^ mice was based on changes in cytokine expression we analyzed the cytokine expression levels of either untreated or treated WT and Ifnar^del^ mice. We chose a set of proinflammatory cytokines (Ifnγ, Tnfα, IL-6, LBP, G-Csf, Ifnα, IL-1α, IL-1β) and anti-inflammatory cytokines (IL-10, IL-13, Tgfβ, Csf) important for the activation of macrophages [33] and performed qRT PCR analysis (Fig 5). Gene expression at mRNA level of caerulein- treated mice revealed a strong upregulation of the proinflammatory cytokines *Tnfα*, *IL-6* and *IL-1β* (Fig 5A) and only a mild increase of the mRNA of the anti-inflammatory cytokines *IL-10*, *Tgfβ1* and *Csf* (Fig 5B) in both genotypes. Interestingly, even untreated Ifnar^del^ mice exhibited extremely high mRNA levels of *Tnfα*, *IL-6*, *IL-1α*, *IL-1β* and *G-Csf* (Fig 5C) and an upregulation of *IL-10 and Csf* (Fig 5D).

### Chemokine upregulation in untreated Ifnar^del^ mice

**Fig 6:**
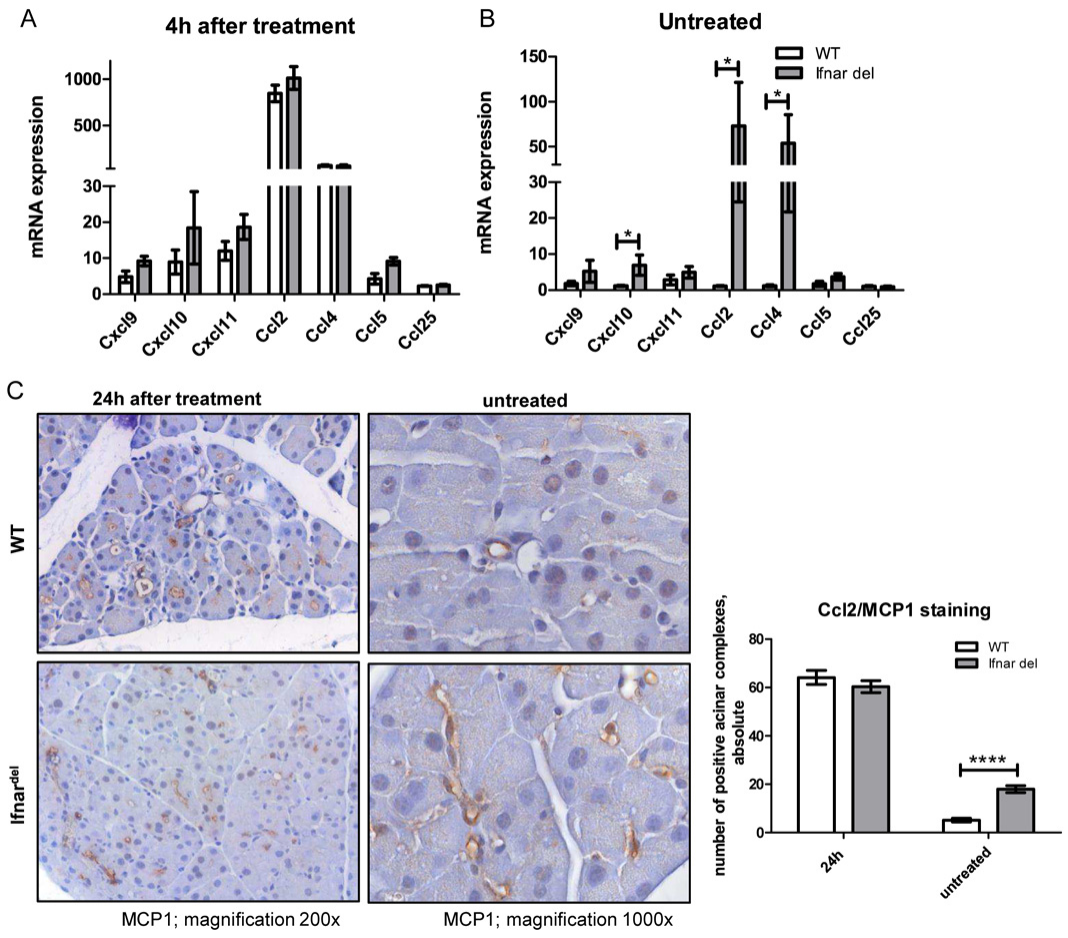
Release of the chemoattractant Ccl2/MCP1 in untreated Ifnar^del^ mice. (A) qRT PCR analysis of mRNA levels of the chemokines *Cxcl9*, *Cxcl10*, *Cxcl11*, *Ccl2*, *Ccl4*, *Ccl5* and *Ccl25* from whole pancreatic tissue of WT and Ifnar^del^ mice harvested 4 hours following caerulein-induced injury (n = 3 per group. Bars indicate mean +/- SD. Normalized on the mRNA of the housekeeping gene *Ppib*). (B) qRT PCR analysis of mRNA levels of the chemokines *Cxcl9*, *Cxcl10*, *Cxcl11*, *Ccl2*, *Ccl4*, *Ccl5* and *Ccl25* from whole pancreatic tissue of untreated WT and Ifnar^del^ mice (n = 7 per group. Bars indicate mean +/- SD. Normalized on the mRNA of the housekeeping gene *Ppib*. *P<0.05, unpaired Student´s t-test). (C) Immunohistochemical staining for MCP1-positive centro-acinar cells in the pancreas from WT and Ifnar^del^ mice 24 hours following caerulein-induced injury or untreated and counting of the absolute number of positive cells on five separate high power fields for each section of untreated WT and Ifnar^del^ mice and after 24 hours following caerulein-induced injury (n = 3–7 per group. Bars indicate mean +/- SD. ****P<0.0001, Mann-Whitney-test. Original magnification, 200x and 1000x).

Since untreated WT and Ifnar^del^ mice showed no persistent macrophages in the pancreatic tissue (S6 Fig), we analyzed whether the early appearance of macrophages in Ifnar^del^ mice 4 h after injury was based on early upregulation of chemokines. After caerulein treatment, both genotypes demonstrated moderate increased levels of *Ccxl9*, *Cxcl10*, *Cxcl11*, *Ccl5*, and *CCcl25* and a distinct upregulation of *Ccl2* and *Ccl4* (Fig 6A). Without treatment, the chemokine expression in WT mice was low whereas the mRNA expression of *Ccxl10* and *Ccl4* was elevated in Ifnar^del^ mice. Moreover, the level of *Ccl2* was extremely high in these mice (Fig 6B). Ccl2, also known as MCP1, is a potent chemotactic factor for monocytes [34]. We performed IHC staining to detect MCP1 in WT and Ifnar^del^ mice to validate our expression data on protein level. 24h after injury there was centroacinar staining in WT as well as in Ifnar^del^ mice (Fig 6C). In untreated WT mice there were no MCP1-positive cells. In contrast, compared to untreated WT mice, Ifnar^del^ mice exhibited an increased expression of MCP1 in the center of the acinar lobes (Fig 6C). The high expression of chemokines and the production of Ccl2/MCP1 in the centro-acinar cells of untreated Ifnar^del^ mice support the hypothesis of an early recruitment of macrophages to the site of inflammation after injury.

## Discussion

In this study, we describe a mouse model limited in type I interferon signaling in pancreatic acini. The restriction of type I interferon signaling in these mice led to a focally limited pancreatitis after caerulein-induced injury with less tissue damage compared to WT mice. Most probably, this observation is due to infiltrating macrophages in this genotype. Indeed, pharmacologic depletion of macrophages reversed this protective effect. Furthermore, we observed that the modification of the interferon signaling increased basal expression levels of cytokines as well as the production of chemokines crucial for the activation and recruitment of macrophages.

Under physiological conditions, type I interferon signaling inhibits the induction of inflammatory cytokines [35, 36]. Indeed, we observed that the expression of cytokines was increased in absence of type I interferon signaling in the pancreas. Similarly, Pinto et al. reported that mice deficient in the interferon receptor express increased levels of proinflammatory cytokines after viral infection [37]. Furthermore, Ifnar^del^ mice also showed an increase of the chemokine Ccl2 in acute pancreatitis. This cytokine-based chemokine release is consistent with the data of Sun et al. showing that acinar cells are responsible for Tnfα-dependent Ccl2 synthesis and secretion [38]. Thereby, our conclusion of an acinar- based cytokine release rather than a release of non acinar cells like endothelial cells or stromal cells is confirmed. Certainly, this boasted release of Ccl2 explains for the significant infiltration of macrophages during the early inflammation phase in Ifnar^del^ mice. Under normal conditions, however, this process of activation and recruitment takes three days [39]. In the current study, we observed that WT mice showed a regular infiltration of regeneration supporting macrophages. However, in Ifnar^del^ mice, we observed an early infiltration of macrophages during the inflammation phase, correlating with increased levels of Ccl2 and decreased tissue damage. This protective effect could be due to the main functions of macrophages: the clearance of dead and damaged cells [16, 40–42] or the support of the resolution of inflammation and tissue regeneration [16, 22]. Our data suggest, that the infiltrating macrophages could phagocytose the damaged cells early on in the course of the inflammation, thereby preventing further release of proinflammatory signals before the inflammation escalates. On the other hand, the infiltrating macrophages could promote an early onset of regeneration thereby limiting the observed tissue damage. Further investigation of the macrophage phenotype are necessary to clarify whether the observed phenotype in Ifnar^del^ mice is based on phagocytic M1, anti-inflammatory M2 macrophages or macrophages in an intermediate state [43].

However, due to the acinar-specific limitation of the interferon signaling, an interferon-dependent reaction of other pancreatic cells could not be considered in the Ifnar^del^ mouse model. Moreover, to exclude an alternative activation of the interferon signaling in acini, a mouse model deficient in the interferon alpha/beta and interferon gamma receptor would be appropriate.

In summary, our data demonstrate that selective loss of Ifnar1 on pancreatic acini results in the enhanced expression of cytokines and production of chemokines already in the untreated mice. This changed environment might lead to priming and a more rapid infiltration of macrophages into the pancreas following injury and confers tissue protection. Thus, type I interferon signaling in pancreatic acinar cells seem to control organ homeostasis by affecting the macrophage-mediated inflammatory response.

## Supporting Information

S1 Table Histological scoring system. Description of the individual histological scoring parameters mentioned grade and distribution of inflammation, edema and necrosis.
(TIF)

S1 Fig Body and pancreas weight of untreated WT and Ifnar^del^ mice. Body weight and pancreas weight of 8 week old WT and Ifnar^del^ mice without treatment (n = 7 per group. Bars indicate mean +/- SD).
(TIF)

S2 Fig ISG stimulation in WT mice after caerulein treatment. qRT PCR analysis of mRNA levels of the interferon-stimulated genes (ISGs) *Dxd58 (RIG-I)*, *Isg15* and *Eifk2ak2 (PKR)* from whole pancreatic tissue of untreated WT mice and 4 h after caerulein treatment. (n = 7 per group. Bars indicate mean +/- SD. Normalized on the mRNA of the housekeeping gene *Ppib*. ***P<0.001, ****P<0.0001, Mann-Whitney-test).
(TIF)

S3 Fig Scoring parameters of pancreatitis evaluation. Individual scoring of pancreatic histological parameters from WT and Ifnar^del^ mice 24, 48 hours, 3, 5, 7 and 14 days following caerulein-induced injury (n = 3–9 per group. Bars indicate mean +/- SD).
(TIF)

S4 Fig Examination of macrophage depletion in WT and Ifnar^del^ mice. Immunohistochemical staining for F4/80-positive macrophages in the pancreas from WT and Ifnar^del^ mice 24 hours following caerulein-induced injury with clodronate based macrophage depletion (upper row) or undepleted (lower row) (n = 3 per group. Original magnification, 200x).
(TIF)

S5 Fig Immune cell compartment in macrophage depleted WT and Ifnar^del^ mice. The absolute number of CD45-positive immune cells, B220-positive B-lymphocytes, CD3-positive T-lymphocytes and MPO-positive neutrophils was counted on five separate high power fields for each section of WT and Ifnar^del^ mice 24 hours following caerulein-induced injury and clodronate based macrophage depletion (n = 3 per group. Bars indicate mean +/- SD).
(TIF)

S6 Fig Macrophage evaluation in untreated WT and Ifnar^del^ mice. Immunohistochemical staining for F4/80-positive macrophages in the pancreas of untreated WT and Ifnar^del^ mice (n = 3 per group. Original magnification, 100x).
(TIF)

S1 Text ARRIVE. Animal Research: Reporting In Vivo Experiments- Checklist
(PDF)
