# Supplementary figures and images for: Loss of Ifnar1 in Pancreatic Acinar Cells Ameliorates the Disease Course of Acute Pancreatitis
Source: PLoS One. 2015 Nov 30;10(11):e0143735. doi: 10.1371/journal.pone.0143735 (PMC4664425; doi:10.1371/journal.pone.0143735)

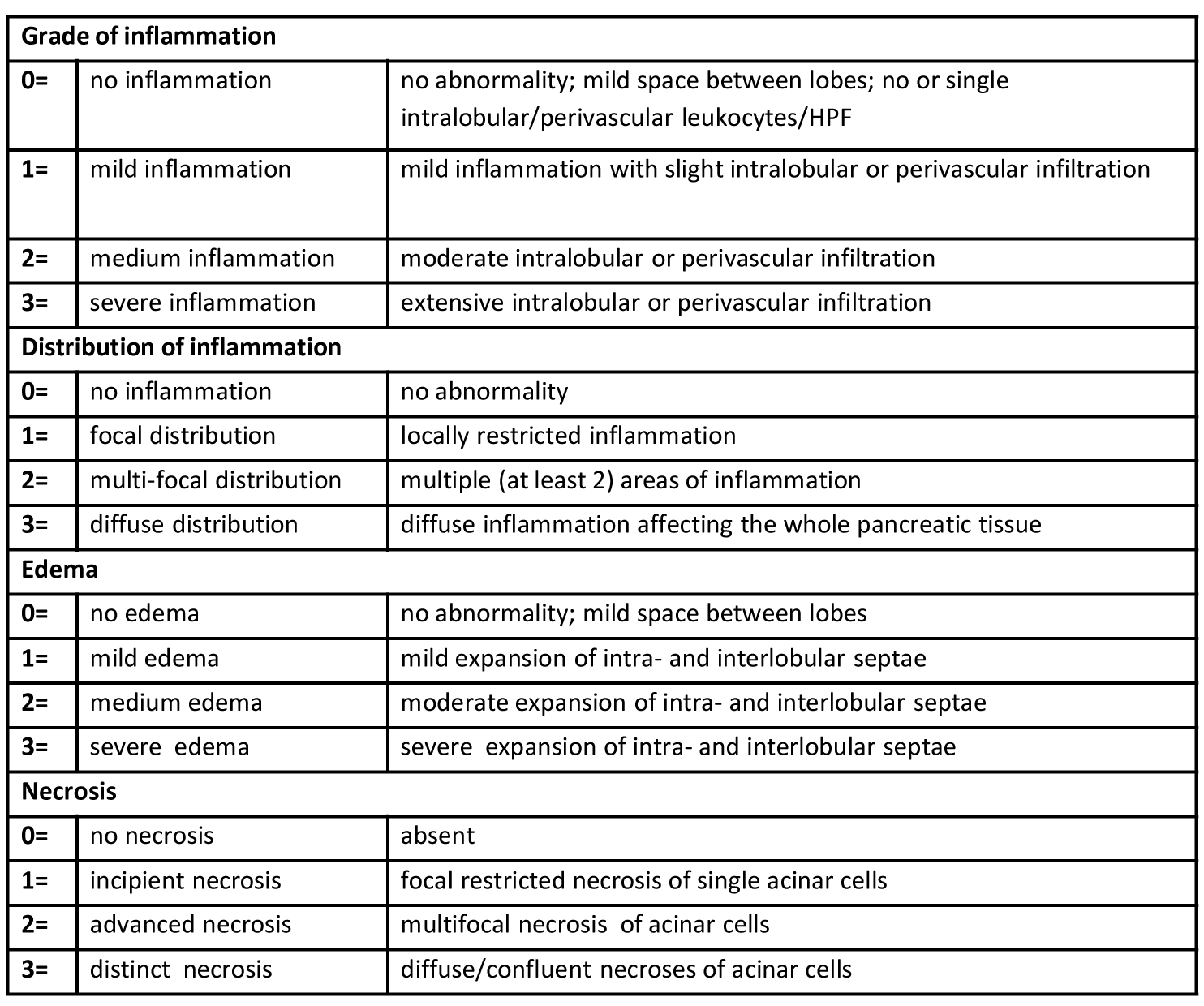

Supplement: S1 Table — Description of the individual histological scoring parameters mentioned grade and distribution of inflammation, edema and necrosis. (TIF) [file pone.0143735.s001.tif]

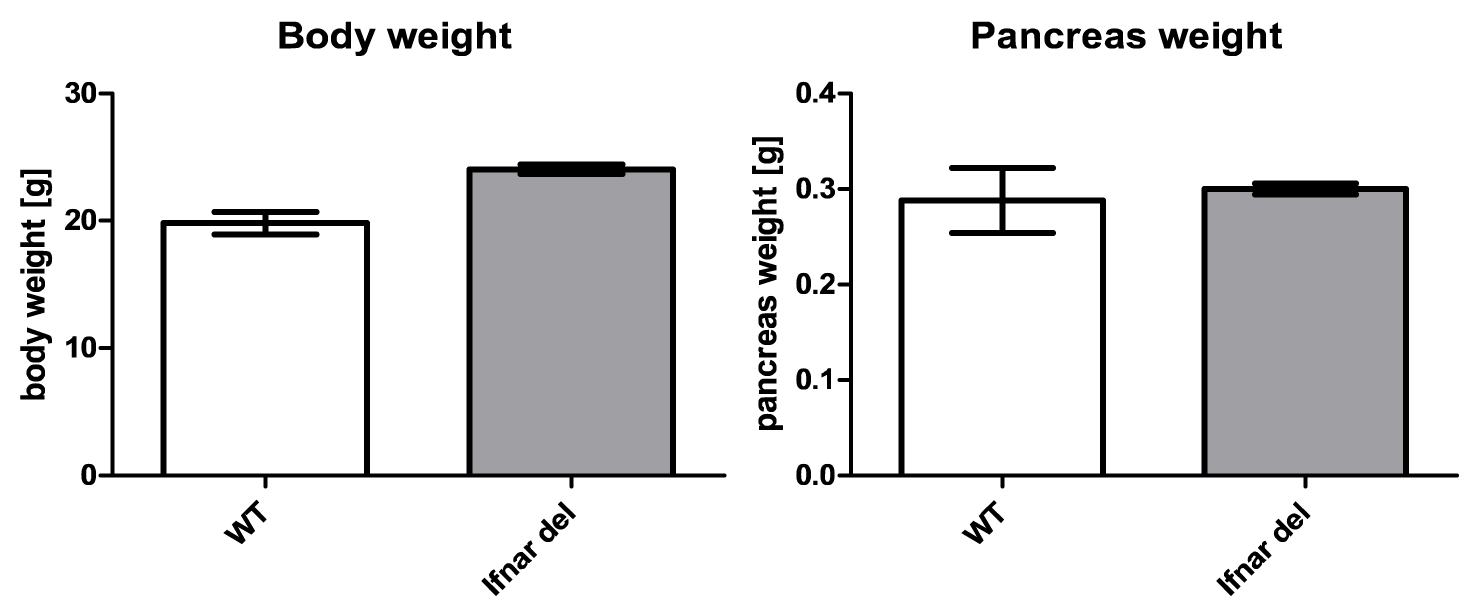

Supplement: S1 Fig — Body weight and pancreas weight of 8 week old WT and Ifnardel mice without treatment (n = 7 per group. Bars indicate mean +/- SD). (TIF) [file pone.0143735.s002.tif]

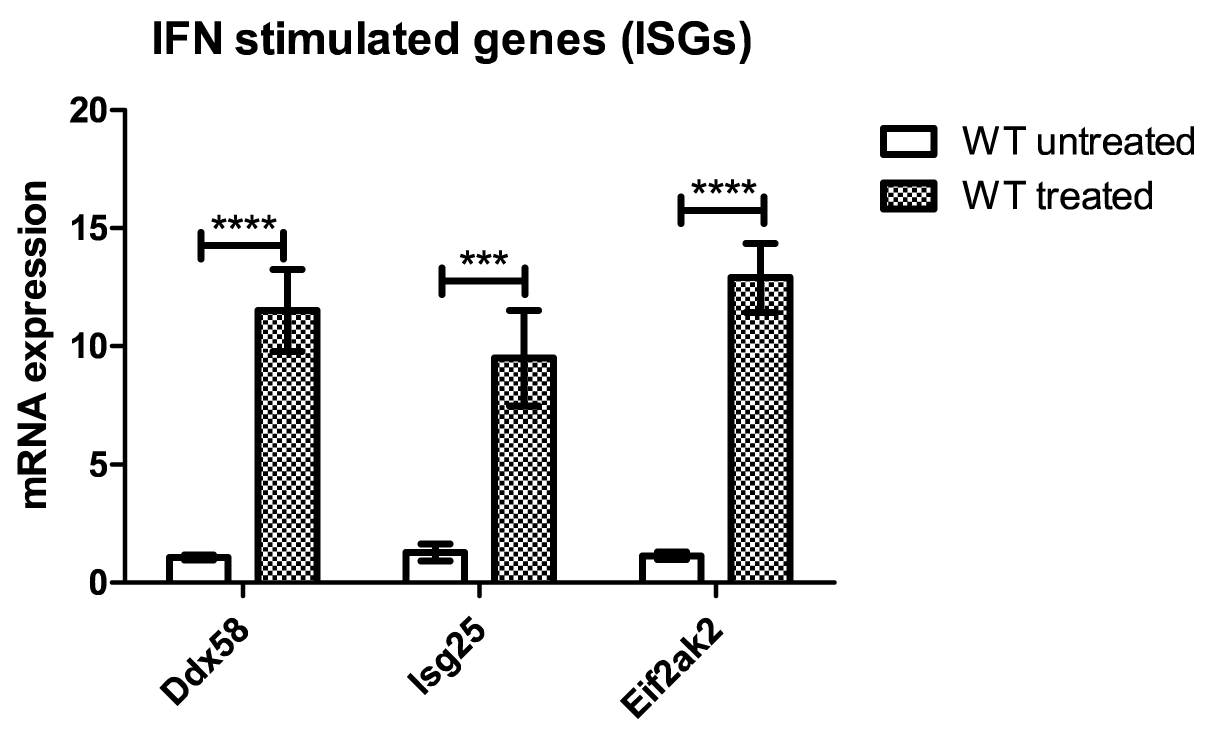

Supplement: S2 Fig — qRT PCR analysis of mRNA levels of the interferon-stimulated genes (ISGs) Dxd58 (RIG-I), Isg15 and Eifk2ak2 (PKR) from whole pancreatic tissue of untreated WT mice and 4 h after caerulein treatment. (n = 7 per group. Bars indicate mean +/- SD. Normalized on the mRNA of the housekeeping gene Ppib. ***P<0.001, ****P<0.0001, Mann-Whitney-test). (TIF) [file pone.0143735.s003.tif]

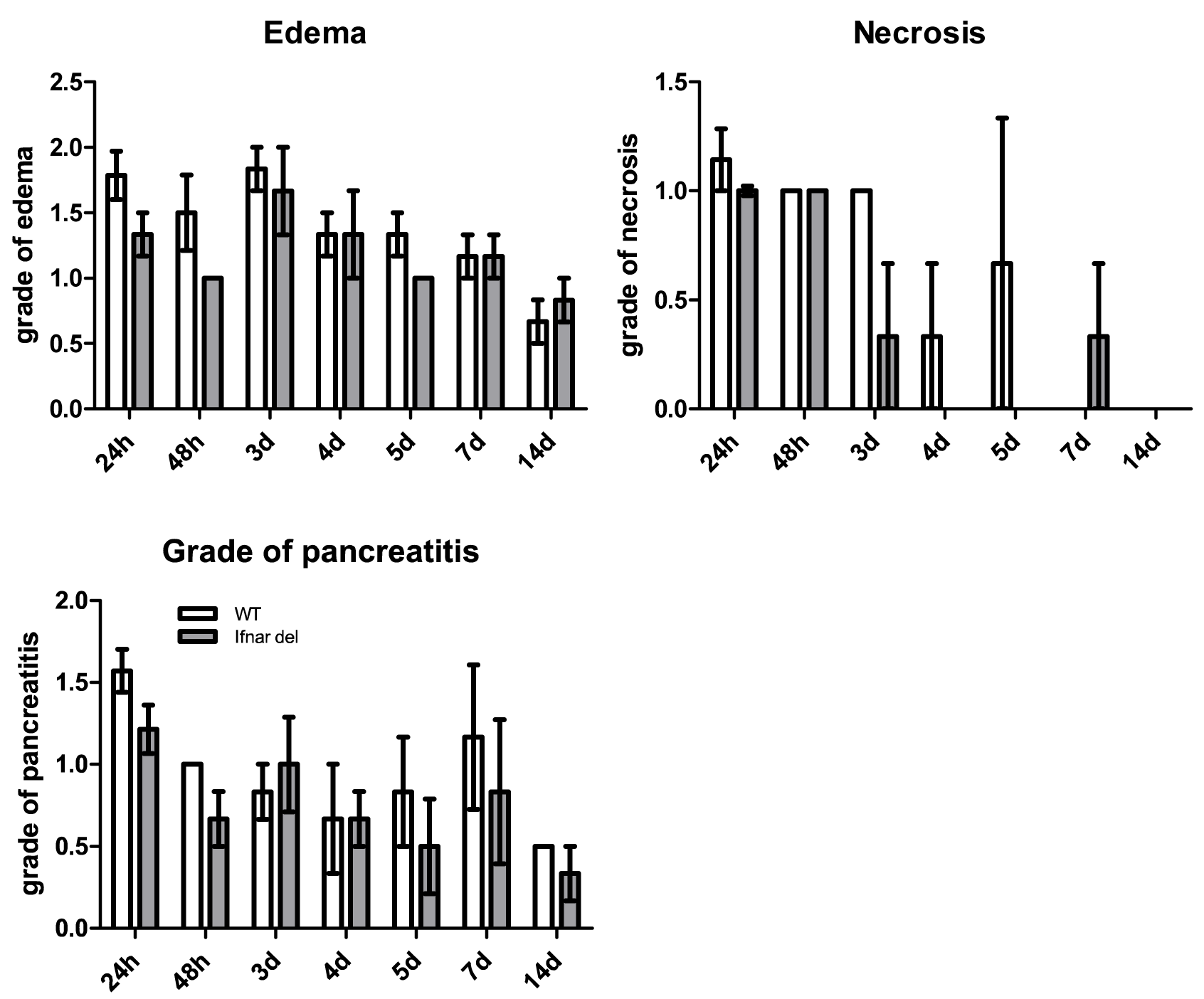

Supplement: S3 Fig — Individual scoring of pancreatic histological parameters from WT and Ifnardel mice 24, 48 hours, 3, 5, 7 and 14 days following caerulein-induced injury (n = 3–9 per group. Bars indicate mean +/- SD). (TIF) [file pone.0143735.s004.tif]

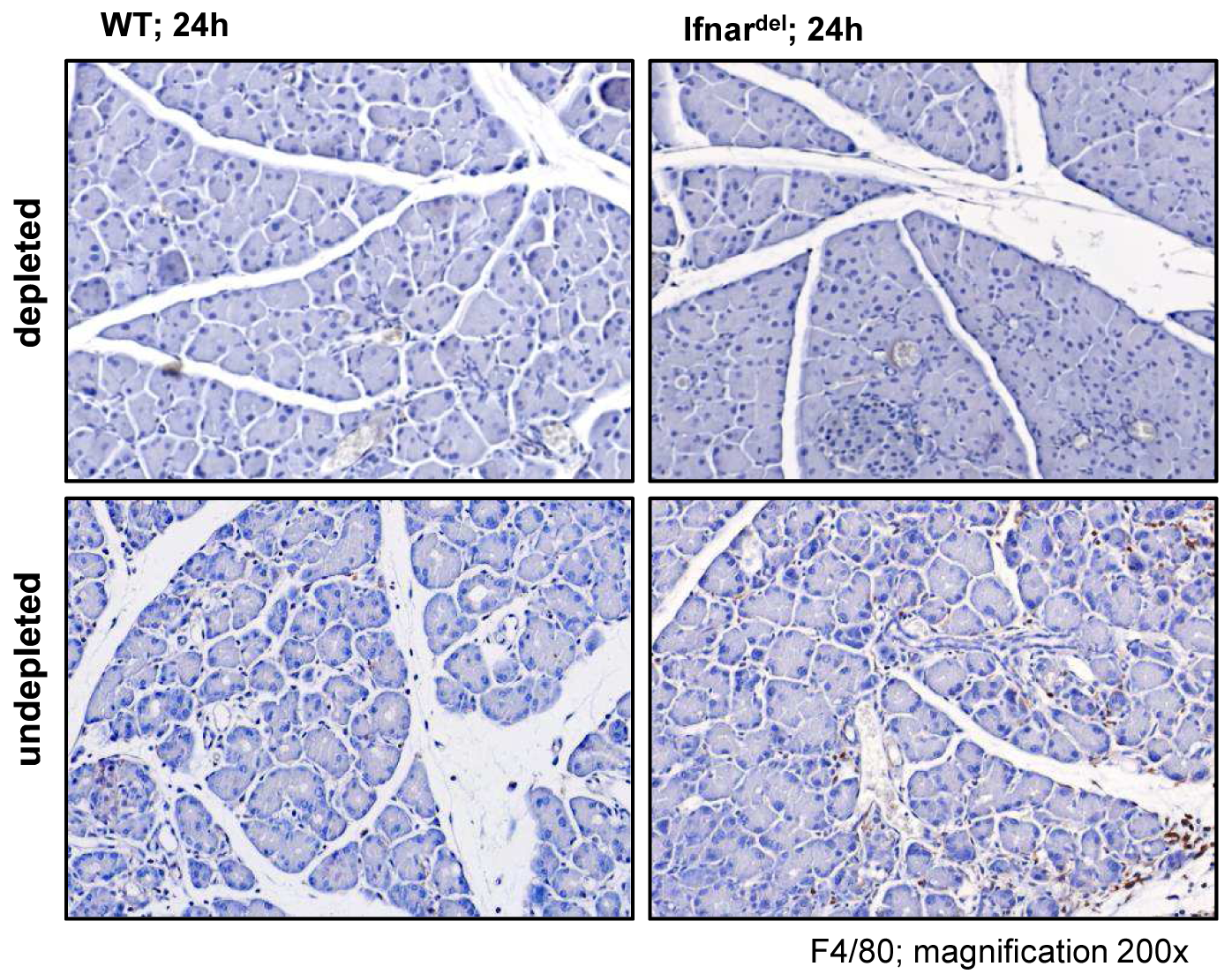

Supplement: S4 Fig — Immunohistochemical staining for F4/80-positive macrophages in the pancreas from WT and Ifnardel mice 24 hours following caerulein-induced injury with clodronate based macrophage depletion (upper row) or undepleted (lower row) (n = 3 per group. Original magnification, 200x). (TIF) [file pone.0143735.s005.tif]

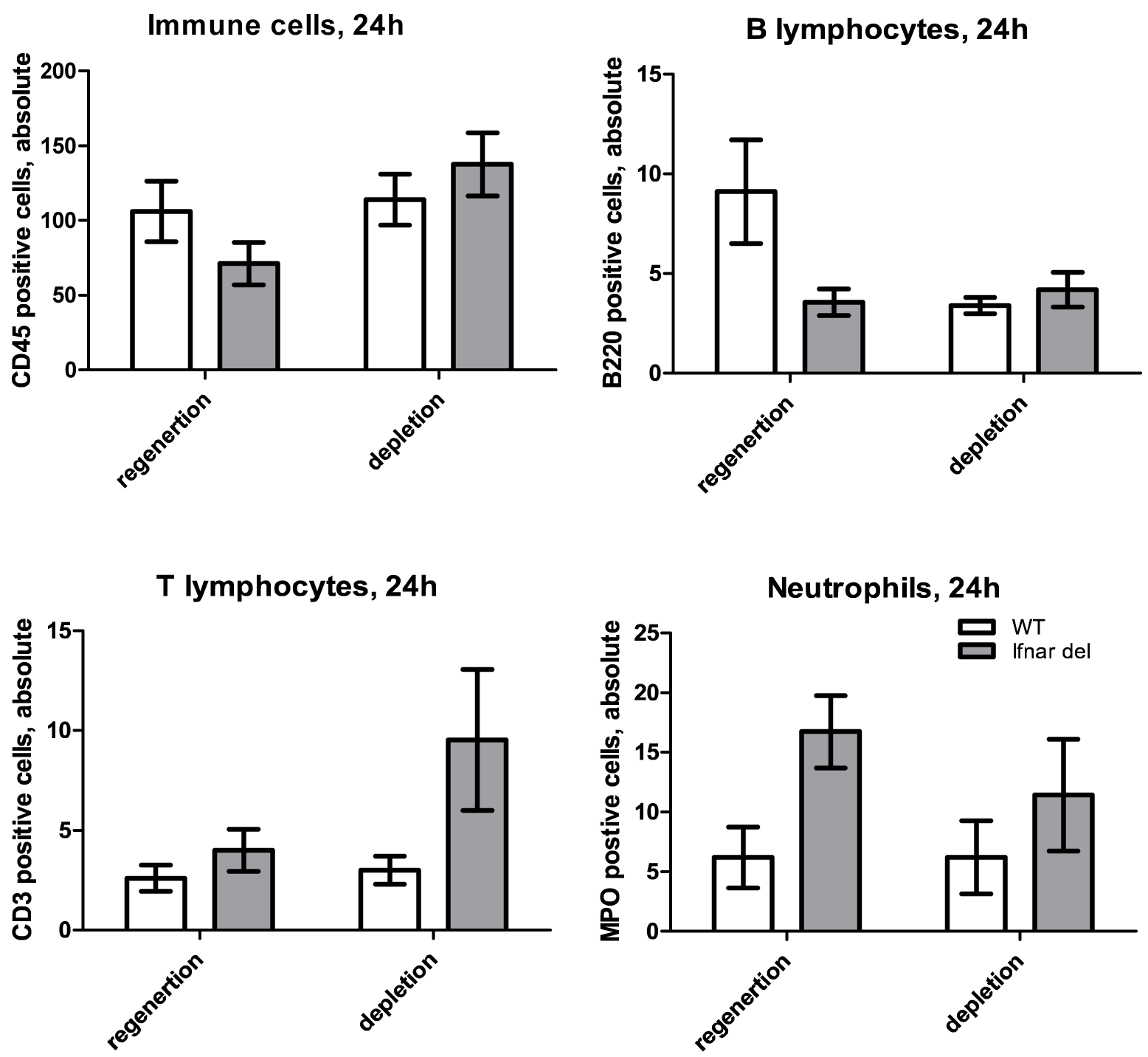

Supplement: S5 Fig — The absolute number of CD45-positive immune cells, B220-positive B-lymphocytes, CD3-positive T-lymphocytes and MPO-positive neutrophils was counted on five separate high power fields for each section of WT and Ifnardel mice 24 hours following caerulein-induced injury and clodronate based macrophage depletion (n = 3 per group. Bars indicate mean +/- SD). (TIF) [file pone.0143735.s006.tif]

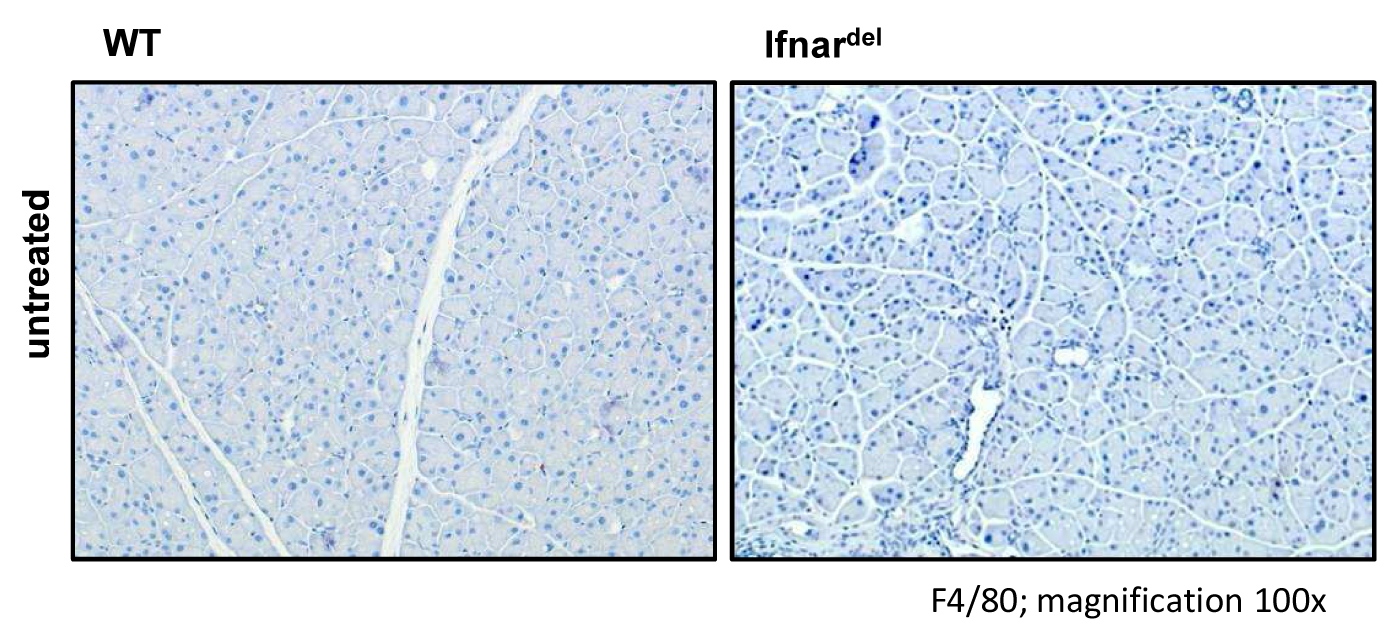

Supplement: S6 Fig — Immunohistochemical staining for F4/80-positive macrophages in the pancreas of untreated WT and Ifnardel mice (n = 3 per group. Original magnification, 100x). (TIF) [file pone.0143735.s007.tif]
